# Sufficient principal component regression for pattern discovery in transcriptomic data

**DOI:** 10.1093/bioadv/vbac033

**Published:** 2022-05-14

**Authors:** Lei Ding, Gabriel E Zentner, Daniel J McDonald

**Affiliations:** Department of Statistics, Indiana University, Bloomington, IN 47405, USA; Department of Biology, Indiana University, Bloomington, IN 47405, USA; Indiana University Melvin and Bren Simon Comprehensive Cancer Center, Indianapolis, IN 46202, USA; Department of Statistics, University of British Columbia, Vancouver, BC, Canada

## Abstract

**Motivation:**

Methods for the global measurement of transcript abundance such as microarrays and RNA-Seq generate datasets in which the number of measured features far exceeds the number of observations. Extracting biologically meaningful and experimentally tractable insights from such data therefore requires high-dimensional prediction. Existing sparse linear approaches to this challenge have been stunningly successful, but some important issues remain. These methods can fail to select the correct features, predict poorly relative to non-sparse alternatives or ignore any unknown grouping structures for the features.

**Results:**

We propose a method called SuffPCR that yields improved predictions in high-dimensional tasks including regression and classification, especially in the typical context of omics with correlated features. SuffPCR first estimates sparse principal components and then estimates a linear model on the recovered subspace. Because the estimated subspace is sparse in the features, the resulting predictions will depend on only a small subset of genes. SuffPCR works well on a variety of simulated and experimental transcriptomic data, performing nearly optimally when the model assumptions are satisfied. We also demonstrate near-optimal theoretical guarantees.

**Availability and implementation:**

Code and raw data are freely available at https://github.com/dajmcdon/suffpcr. Package documentation may be viewed at https://dajmcdon.github.io/suffpcr.

**Contact:**

daniel@stat.ubc.ca

**Supplementary information:**

Supplementary data are available at *Bioinformatics Advances* online.

## 1 Introduction

Global transcriptome measurement with microarrays and RNA-Seq is a staple approach in many areas of biological research and has yielded numerous insights into gene regulation. Given data from such experiments, it is often desirable to identify a small number of transcripts whose expression levels are associated with a phenotype of interest (for instance, disease-free survival of cancer patients). Indeed, projects such as The Cancer Genome Atlas have aimed to generate massive volumes of such data to enable molecular characterization of various cancers. While these data are readily available, their high-dimensional nature (tens of thousands of transcript measurements from a single experiment) makes identification of a compact gene expression signature statistically and computationally challenging. While the identification of a minimal gene expression signature is valuable in evaluating disease prognosis, it is also helpful for guiding experimental exploration. In practical terms, a set of five genes highly associated with a certain disease phenotype can be characterized more rapidly, at lower cost, and in more depth than a set of 50 or 100 such genes using genetic techniques such as CRISPR knockout and cancer biological methods such as xenotransplantation of genetically modified cells into mice. Therefore, this article prioritizes selecting a small subset of transcript measurements, which still provide an accurate prediction of phenotypes.

With these goals in mind, supervised linear regression techniques such as ridge regression (Hoerl and Kennard, 1970), the lasso (Tibshirani, 1996), elastic net (Zou and Hastie, 2005) or other penalized methods are often employed. More commonly, especially in genomics applications, the outcomes of interest tend to be the result of groups of genes, which perhaps together describe more complicated processes. Therefore, researchers often turn to unsupervised methods such as principal component analysis (PCA), principal component regression (PCR) and partial least squares (PLS) for both preprocessing and as predictive models (e.g. Cera *et al.*, 2019; Harel *et al.*, 2019; Kabir *et al.*, 2017; Traglia *et al.*, 2017).

In genomics, one may collect expression measurements for thousands of genes from microarrays or RNA-Seq with the goal of predicting phenotypes or class outcomes. In these settings, the number of patients is much smaller than the number of gene measurements and researchers are interested in (i) the accurate prediction of the phenotype, (ii) the correct identification of a handful of predictive genes and (iii) computational tractability. Among these properties, the correct identification of a small number of predictive genes is of crucial importance in practice, since it can lead biologists to further investigate specific genes through CRISPR knockout or other techniques. It is this genetic pattern discovery for which our proposed methodology is intended: data with many more measurements than observations; the potential that some of the measurements may be grouped or correlated; the existence of either a continuous or discrete outcome we wish to predict; and the belief that these predictions only depend on some small collection of groups rather than the entire set of measurements.

### 1.1 Recent related work

PCA has two main drawbacks when used in high dimensions. The first is that PCA is non-sparse, so it uses information from all the available genes instead of selecting only those which are important, a key objective in omics applications. That is, the right singular vectors or ‘eigengenes’ (Alter *et al.*, 2000) depend on all the genes measured rather than a small collection. The second is that these sample principal components are not consistent estimators of the population parameters in high dimensions (Johnstone and Lu, 2009). This means essentially that when the number of patients is smaller than the number of genes, even if the first eigengene could perfectly explain the data, PCA will not be able to recover it.

Modern approaches specifically for pattern discovery in the genomics context such as supervised gene shaving (Hastie *et al.*, 2000), tree harvesting (Hastie *et al.*, 2001) and supervised principal components (SPC) (Bair and Tibshirani, 2004; Bair *et al.*, 2006; Paul *et al.*, 2008) seek to combine the presence of the phenotype with the structure estimation properties of eigendecompositions on the gene expression measurements using unsupervised techniques to obtain the best of both. PLS is common in genomics (e.g. Chakraborty, 2019; Leek and Storey, 2007), though it remains uncommon in statistics and machine learning, and its theoretical properties are poorly understood. Other recent PCA-based approaches for genetics, though not directly applicable for prediction are SMSSVD (Henningsson and Fontes, 2019) and ESPCA (Min *et al.*, 2018).

### 1.2 Contributions

In this paper, we leverage the strong theoretical properties associated with sparse PCA to improve predictive accuracy for regression and classification problems in genomics. We avoid the strong assumptions necessary for SPC, the current state-of-the-art, while obtaining the benefits associated with sparse subspace estimation. In the case that the phenotype is actually generated as a linear function of a handful of genes, our method, SuffPCR, performs nearly optimally: it does as well as if we had known which genes were relevant beforehand. Furthermore, we justify theoretically that our procedure can both predict accurately and recover the correct genes. Our contributions can be succinctly summarized as follows:

1. We present a methodology for discovering small sets of predictive genes using sparse PCA;
2. Our method improves the computational properties of existing sparse subspace estimation approaches to enable previously impossible inference when the number of genes is very large;
3. We demonstrate state-of-the-art performance of our method in synthetic examples and with standard cancer microarray measurements;
4. We provide near-optimal theoretical guarantees.

Our methodology can be used in a variety of genomic pattern discovery settings. One such example is a modified version of traditional differential expression analysis. If we have treatment and control measurements, the logistic version of our method is appropriate with the advantage that it examines the impact of one gene adjusted for the contributions of others. In addition, with a continuous treatment, the detection power can be increased relative to using an artificial dichotomization.

In Section 2.1, we motivate the desire for *sufficient* PCR relative to previous approaches and present details of SuffPCR. Section 2.2 illustrates performance in simulated, semi-simulated and real examples (Section 2.3) and discusses the biological implications of our methods for a selection of cancers. Section 2.4 theoretically justifies our methods, providing guarantees for prediction accuracy and correct gene selection. Section 3 concludes.

*Notation*. We use bold uppercase letters to denote matrices, lowercase Arabic letters to denote row vectors and scalars and uppercase Arabic letters for random variables. Let *Y* be a random, real-valued *n*-vector of independent variables *Y_i_*, and **X** be the row-wise concatenation of i.i.d. draws *X_i_* from a distribution on $R^{p}$ with covariance Σ. We denote the observed realization of the outcome variable *Y* as $y\in R^{n}$. To be explicit in the genomics context, **X** is an *n *×* p* matrix where each row is a set of transcriptomic measurements from RNA-Seq or microarrays for a patient while *y_i_* is an observed phenotype of interest for the *i*th patient. Because **X** is a matrix, this symbol represents both a random matrix and its realization. In the following, the meaning should be clear from the context. We assume, without loss of generality, that $E\left[X_{i}\right]=0$ and that the measurements **X** have been centered. The singular value decomposition of a matrix **A** is $A=U(A)\Lambda (A)V^{T}(A)$. In the specific case of **X**, we suppress the dependence on **X** in the notation and write $X=U\Lambda V^{T}$. We write $A_{d}$ to indicate the first *d* columns of the matrix **A** and *a_j_* to denote the *j*th row. In the case of the identity matrix, we use a subscript to denote its dimension when necessary: $I_{p}$. Let tr(A) denote the sum of the diagonal entries of **A** while $||A|{|}_{F}^{2}=\sum_{ij}a_{ij}^{2}$ is the squared Frobenius norm of **A**. $||A|{|}_{2,0}$ denotes (2, 0)-norm of **A**, that is the number of rows in **A** that have non-zero $\ell_{2}$ norm. $||A|{|}_{1,1}$ is the sum of the row-wise $\ell_{1}$ norms. Finally, 1(a) is the indicator function for the expression *a*, taking value 1 if *a* is true or 0 if not.

## 2 Methods

SPC (Bair and Tibshirani, 2004; Bair *et al.*, 2006; Paul *et al.*, 2008) is widely used for solving high-dimensional prediction and feature selection problems. It targets dimension reduction and sparsity simultaneously by first screening genes [or individual messenger RNA (mRNA) probes] based on their marginal correlation with the phenotype (or likelihood ratio test in the case of non-Gaussian noise). Then, it performs PCA on this selected subset and regresses the phenotype on the resulting components (possibly with additional penalization). This procedure is computationally simple, but, zero population marginal correlation is neither necessary nor sufficient to guarantee that the associated population regression coefficient is zero. To make this statement mathematically precise, consider the linear model $Y_{i}=X_{i}^{T}\beta^{*}+\epsilon_{i},$ where *Y_i_* is a real-valued scalar phenotype, *X_i_* is a real-valued vector of genes, $\beta^{*}$ is the true (unknown) coefficient vector and *ϵ_i_* is a mean-zero error. Defining as above $\text{Cov}(X_{i},X_{i})=\Sigma$, and $\text{Cov}(X_{i},Y_{i})=\Phi$, then, for this procedure to correctly recover the true nonzero components of $\beta^{*}$, it requires

(1) $$\Phi_{j}=0\Rightarrow \beta_{j}^{*}={\left(\Sigma^{-1}\Phi \right)}_{j}=0.$$

In words, we assume that the dot product of the *j*th row of the precision matrix with the marginal covariance between *x* and *y* is zero whenever the *j*th element of Φ is zero. While reasonable in some settings, this assumption frequently fails. For example, individual features may only be predictive of the response in the presence of other features. To illustrate why this assumption fails for genomics problems, we examine a motivating counterexample. Using mRNA measurements for acute myeloid leukemia (AML, Bullinger *et al.* 2004), we estimate both $\Sigma^{-1}$ and Φ and proceed as if these estimates are the true population quantities. To estimate Φ, we use the empirical covariance and set all but the largest *n *=* *116 values equal to zero, corresponding to an extremely sparse estimate. For $\Sigma^{-1}$, we use the Graphical Lasso (Friedman *et al.*, 2008) for all *p *=* *6283 genes at different sparsity levels ranging from 100% sparse (${\hat{\Sigma }}_{ij}^{-1}=0$ for all i≠j) to 95% sparse. We then create a pseudotrue $\beta^{*}={\hat{\Sigma }}^{-1}\hat{\Phi }$ as in Equation (1). This is essentially the most favorable condition for SPC. To reiterate, in order to evaluate this assumption, we create $\beta^{*}$ based on estimates from real genetics data that are highly sparse. But, as we will see below, because the inverse covariance matrix is not ‘sparse in the right way’, SPC will have a very high false negative rate and ignore important genes.

Table 1 shows the sparsity of ${\hat{\Sigma }}^{-1}$, the percent of non-zero regression coefficients, and the percent of non-zero regression coefficients which are incorrectly ignored under the assumption (the false negative rate). Even if the precision matrix is 99.9% sparse, the false negative rate is over 40%, meaning we find fewer than 60% of the true genes. If the sparsity of ${\hat{\Sigma }}^{-1}$ is allowed to decrease only slightly, the false negative rate increases to over 95%. Clearly, this screening procedure will ignore many important genes in even the most favorable conditions for SPC.

**Table 1.** Illustration of the failure of Equation (1) on the AML data

% sparsity of ${\hat{\Sigma }}^{-1}$	100	99.9	99.6	98.9	97.5	95.3
% non-zero $\beta^{*}$’s	1.8	3.3	8.4	23.5	50.2	77.9
False negative rate	0.000	0.431	0.778	0.921	0.963	0.976

More recent work has attempted to avoid this assumption. Ding and McDonald (2017) uses the initially selected set of features to approximate the information lost in the screening step via techniques from numerical linear algebra. An alternative discussed in Piironen and Vehtari (2018) iterates the screening step with the prediction step, adding back features which correlate with the residual. Finally, Tay *et al.* (2018) assumes that feature groupings are known and and estimates separate subspaces for different groups. All these methodologies are tailored to perform well when Φ and $\beta^{*}$ have particular compatible structures.

On the other hand, it is important to observe that a sufficient condition for $\beta_{j}^{*}=0$ in Equation (1) is that the *j*th row of the left eigenvectors of Σ is 0. Based on this intuition, we develop sufficient PCR (abbreviated as SuffPCR) which leverages this insight: row sparse eigenvectors imply sparse coefficients, and hence depend on only a subset of genes. SuffPCR is tailored to the case that **X** lies approximately on a low-dimensional linear manifold which depends on a small subset of features. Because the linear manifold depends on only some of the features, $\beta^{*}$ does as well.

### 2.1 Prediction with principal components

PCA is a canonical unsupervised dimension reduction method when it is reasonable to imagine that **X** lies on (or near) a low-dimensional linear manifold. It finds the best *d*-dimensional approximation of the span of **X** such that the reconstruction error in $\ell_{2}$ norm is minimized. This problem is equivalent to maximizing the variance explained by the projection:

(2) $$\underset{V}{\max}\;\text{tr}\left(SVV^{T}\right)\;\text{subject to}\;V^{T}V=I_{d},$$

where $S=\frac{1}{n}X^{T}X$ is the sample covariance matrix. Let $X=U\Lambda V^{T}$, then the solution of this optimization problem is $V_{d}$, the first *d* right singular vectors, and the estimator of the first *d* principal components is $U_{d}\Lambda_{d}$ or $XV_{d}$ equivalently. Given an estimate of the principal components, PCR is simply ordinary least squares (OLS) regression of the phenotype on the derived components $U_{d}\Lambda_{d}$. One can convert the lower-dimensional estimator, say $\hat{\gamma }$, back to the original space to reacquire an estimator of $\beta^{*}$ as $\beta^{*}$. Other generalized linear models can be used place of OLS to find $\hat{\gamma }$.

#### 2.1.1 Sparse principal component analysis

As discussed in Section 1.1, standard PCA works poorly in high dimensions. Much like the high-dimensional regression problem, estimating high-dimensional principal components is ill-posed without additional structure. To address this issue many authors have focused on different sparse PCA estimators for the case when **V** is sparse in some sense. Many of these methods achieve this goal by adding a penalty to Equation (2). Of particular utility for the case of PCR when $\beta^{*}$ is sparse is to choose a penalty that results in row-sparse **V**. This intuition is justified by the following result.

Proposition 1. *Consider the linear model* $Y_{i}=X_{i}^{T}\beta^{*}+\epsilon$*with* $\text{Cov}(X_{i},X_{i})=\Sigma$*. Let* $\Sigma =V(\Sigma )\Lambda (\Sigma )V{\left(\Sigma \right)}^{T}$*be the eigendecomposition of* Σ*with* $\Lambda {\left(\Sigma \right)}_{jj}=0$*for* $j>d\in Z^{+}$*. Then* $||v{\left(\Sigma \right)}_{j}|{|}_{2}=0\Rightarrow \beta_{j}^{*}=0.$

The proof is immediate. For any *j*, if $||v{\left(\Sigma \right)}_{j}|{|}_{2}=0$, then every element in $v{\left(\Sigma \right)}_{j}$ is 0, indicating the $j^{\text{th}}$ row of $\Sigma^{-1}$ will be 0. Since $\beta_{j}^{*}={\left(\Sigma^{-1}\Phi \right)}_{j}$ where $\text{Cov}(X_{i},y_{i})=\Phi$, it also results in $\beta_{j}^{*}=0$. This result stands in stark contrast to the assumption in Equation (1). This proposition gives a guarantee rather than requiring an assumption: if the rows of $V_{d}$ are sparse, then $\beta_{*}$ is sparse. The same intuition can easily be extended to the case $\Lambda {\left(\Sigma \right)}_{jj}\geq 0$ for all *j* given a gap between the *d*th and ${\left(d+1\right)}^{\text{st}}$ eigenvalues. In this setting, the natural analogue of PCA is the solution to:

(3) $$\underset{V}{\max}\;\text{tr}\left(SVV^{T}\right)-\lambda {\left\|V\right\|}_{2,0}^{2}\;\text{subject to}\;V^{T}V=I_{d}.$$

Solutions ${\hat{V}}_{d}$ of Equation (3) will give projection matrices onto the best *d*-dimensional linear manifold such that ${\hat{V}}_{d}$ is row sparse. However, this problem is NP-hard.

Many authors have developed different versions of sparse PCA. For example, d’Aspremont *et al.* (2005) and Zou *et al.* (2006) focus on the first principal component and add additional principal components iteratively to account for the variation left unexplained by the previous principal components. Vu and Lei (2013) derive a rate-minimax lower bound, illustrating that no estimator can approach the population quantity faster than, essentially, $q\sqrt{d/n}$ where *q* is a deterministic function of Σ. Later work in Vu *et al.* (2013) proposes a convex relaxation to Equation (3) which finds the first *d* principal components simultaneously and nearly achieves the lower bound:

(4) $$\underset{V}{\max}\;\text{tr}\left(SVV^{T}\right)-\lambda ||VV^{T}|{|}_{1,1}\;\text{subject to}\;VV^{T}\in F^{d},$$

where $F^{d}:=\{VV^{T}:0\;⪯\;VV^{T}\;⪯\;I_{p}\;\text{and tr}(VV^{T})=d\}$ is a convex body called Fantope, motivating the name Fantope Projection and Selection (FPS). The authors solve the optimization problem in Equation (4) with an alternating direction method of multipliers (ADMM) algorithm.

For these reasons, FPS is known as the current state-of-the-art sparse PCA estimator with the best performance. However, despite its theoretical justification, FPS is less useful in practice for solving prediction tasks, especially in genomics applications with p≫n (rather than just *p *>* n*) for two reasons. First, the original ADMM algorithm has per-iteration computational complexity $O(p^{3})$, which is a burden especially when *p* is large. Second, because of the convex relaxation using Equation (4) rather than Equation (3), ${\hat{V}}_{d}$ from FPS tends to be entry-wise sparse, but infrequently row-wise sparse unless the signal-to-noise ratio (SNR) is very large (*q* is a function of this ratio). We give explicit formulas for the SNR under this model in the Supplementary Material, but heuristically, the SNR captures how well the data is described by a *d*-dimensional subspace through the relative magnitude of $\text{tr}\left(\Lambda_{d}\right)$ compared to *p*. In genomics applications with low SNR, which is common, estimates $\hat{\beta }$ tend to have large numbers of non-zero coefficients with very small estimated values. Thus, we design SuffPCR based on the insights from Proposition 1, utilizing the best sparse PCA estimator FPS and further addressing both of these issues to achieve better empirical performance while maintaining theoretical justification.

#### 2.1.2 Sufficient principal component regression

**Fig. 1.**
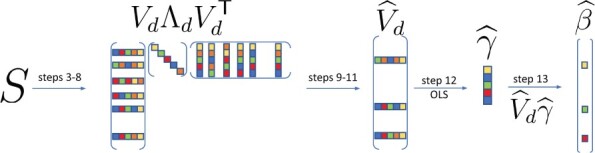
Graphical depiction of Algorithm 1. Solid colors represent nonzero matrix entries

In this section, we introduce SuffPCR. The main idea of SuffPCR is to detect the relationship between the phenotype *Y* and gene expression measurements **X** by making use of the (near) low-dimensional manifold that supports **X**. In broad outline, SuffPCR first uses a tailored version of FPS to produce a row-sparse estimate ${\hat{V}}_{d}$ and then regresses *Y* on the derived components to produce sparse coefficient estimates. SuffPCR for regression is stated in Algorithm 1 and summarized visually in Figure 1. For ease of exposition, we remind the reader that *Y* and **X** are standardized so that $S=\frac{1}{n}X^{T}X$ is the correlation matrix.

Algorithm 1 SuffPCR (regression version)

1: **Input: X**, **S**, *y*, *d*, *λ*.

2: B←0,C←0    ▹ Initialization

3: **while** not converged **do**

4: $A\leftarrow {\text{Proj}}_{F^{d}}\left(B-C+S/\lambda \right)$ ▹ Approximate projection

5: B←Soft(A+C)       ▹ Elementwise soft-thresholding

6: C←C+A−B

7: **end while**

8: Decompose $B=V_{d}\Lambda_{d}V_{d}^{T}$ ▹ Rank *d* eigen decomposition

9: Compute $l=\text{diag}(V_{d}V_{d}^{T})$, sort in descending order

10: Choose *t* by applying Algorithm 2 to *l*

11: Set rows in $V_{d}$ whose $\ell_{2}$ norm is smaller than *t* as 0, and get ${\hat{V}}_{d}$

12: Solve $\hat{\gamma }={\text{argmin}}_{\gamma }||y-X{\hat{V}}_{d}\gamma |{|}_{2}^{2}$

13: **Return:**$\hat{\beta }={\hat{V}}_{d}\hat{\gamma }$

The first issue is the time complexity of the original FPS algorithm. Essentially, FPS uses the same three steps depicted in Lines 4–6 in Algorithm 1.

4′. $A\leftarrow {\text{Proj}}_{F^{d}}\left(B-C+S/\lambda \right)$

5. B←Soft(A+C) where Soft(b)=sign(b)max{|b|−1, 0}

6. C←C+A−B.

The only difference here between our implementation and that in FPS is in Step 4. Each of these steps takes a matrix and produces another matrix, where the matrices have *p*^2^ elements. The second and third steps are computationally simple (element-wise soft-thresholding and matrix addition). But the first, ${\text{Proj}}_{F^{d}}(Q)$, is more challenging. The solution requires computing the eigendecomposition of **Q**, an $O(p^{3})$ operation, and then modifying the eigenvalues of **Q** through the solution of a piecewise linear equation in *τ*: $\Lambda_{i,+}^{2}(Q)=\min\{\max\{\Lambda_{i}^{2}(Q)-\tau ,\;0\},\;1\},$ with *τ* such that $\sum_{i=1}^{\min\{n,p\}}\Lambda_{i,+}^{2}(Q)=d$. The final result is then reconstructed as $A=U(Q)\Lambda_{+}^{2}(Q)U{\left(Q\right)}^{T}$. Because of the cubic complexity in *p*, the authors suggest the number of features should not exceed one thousand. But typical transcriptomics data have many thousands of gene measurements, and preliminary selection of a subset is suboptimal, as illustrated above. Due to the form of the piecewise solution, most eigenvalues will be set to 0. Thus, while we will generally require more than *d* eigenpairs, most are unnecessary, certainly fewer than min{n,p}. Our implementation computes only a handful of eigenvectors corresponding to the largest eigenvalues, rather than all *p*. If we compute enough to ensure that some $\Lambda_{i,+}^{2}(Q)$ will be 0, then the rest are as well. Our implementation uses Augmented Implicitly Restarted Lanczos Bidiagonalization (AIRLB; Baglama and Reichel, 2005) as implemented in the irlba package (Baglama *et al.*, 2019), though alternative techniques such as those in Homrighausen and McDonald (2016); Gittens and Mahoney (2013) may work as well. We provide a more detailed discussion in the Supplementary Material.

For moderate problems (n,p≈100), the truncated eigendecomposition with AIRLB rather than the full eigendecomposition leads to a three-fold speedup while the further incorporation of specialized initializations leads to an eight-fold improvement without any discernable loss of accuracy (results on a 2018 MacBook Pro with 2.7 GHz Quad-Core processor and 16GB of memory running maxOS 10.15). The results are similar when *p *=* *5000, though the same experiment on a high-performance Intel Xeon E5-2680 v3 CPU with 12 cores, 256 GB of memory, and optimized BLAS were somewhat less dramatic (improvements of three-fold and four-fold respectively). For large RNA-Seq datasets (p≈20 000), we observed a nearly ten-fold improvement in computation time.

The second issue is that the Fantope constraint in Equation (4) ensures only that $\text{tr}(VV^{T})=d$ but not that the number of rows with non-zero *l*_2_-norm is small. This feature of the convex relaxation results in many rows with small, but non-zero, row-norm resulting in dense estimates of $\beta^{*}$. Thus, to make the final estimator ${\hat{V}}_{d}$ sparse, we hard-threshold rows in ${\hat{V}}_{d}$ whose $\ell_{2}$ norm is small, as illustrated in line 9, 10 and 11 in Algorithm 1. From empirical experience, we have found that there is often a strong elbow-type behavior in the row-wise $\ell_{2}$ norm of ${\hat{V}}_{d}$, similar to the Skree plot used to choose *d* in standard PCA. Therefore, we develop a simple procedure, Algorithm 2, to find the best threshold automatically. Essentially, it calculates the empirical derivative of the observation-weighted variances on each side of a potential threshold and maximizes their difference, resulting in signal and noise groups. We set the rows in ${\hat{V}}_{d}$ corresponding to the noise to 0. SuffPCR is also amenable for solving other generalized linear models. For example, replacing line 12 in Algorithm 1 with logistic regression solves classification problems.

Algorithm 2 Find a *t* to hard-threshold *l*

1: **Input:** a *p*-vector *l*

2: **for**i∈1,…,p**do**

3: $T_{n}[i]=\mathrm{var}(l[1:i])$

4: $T_{s}[i]=\mathrm{var}(l[(i+1):p])$

5: $T[i]=i*T_{n}[i]+(p-i)T_{s}[i]$

6: δ[i]=T[i]−T[i−1]    ▹ empirical derivative of *T*

7: **end for**

8: Set $i^{*}={\text{argmin}}_{i}\{\delta [i]-\delta [i-1]>\mathrm{mean}(|\delta [1:(i-1)]|)\}$

9: **Return:**$t=l[i^{*}]$

### 2.2 Synthetic data experiments

In this section, we show how SuffPCR performs on synthetic data and on real public genomics datasets relative to state-of-the-art methods. Section 2.2.1 first presents a generative model for synthetic data and motivates the assumptions required for our theoretical results in Section 2.2.4. We include here one synthetic experiment under conditions favorable to SuffPCR relative to SPC. We also investigate conditions favorable to SPC, the influence of tuning parameter selection, and the effect of the signal to noise ratio but defer these to the Supplementary Material. Section 2.2.3 uses the non-small-cell lung cancer (NSCLC) data as the **X** matrix but creates the response from a linear model. Section 2.3 reports the performance of SuffPCR on 5 public genomics datasets. The Supplementary Material includes similar results for binary survival-status outcomes. Across most settings in both synthetic and real data, SuffPCR outperforms all competitors in prediction mean-squared error and is able to select the true genes (those with $\beta^{*}\neq 0$) more accurately. An R package implementing SuffPCR and raw data are freely available at https://github.com/dajmcdon/suffpcr. Package documentation may be viewed at https://dajmcdon.github.io/suffpcr.

#### 2.2.1 Experimental setup

We generate data from the multivatiate Gaussian linear model $y_{i}=x_{i}^{T}\beta^{*}+\epsilon_{i},$ where $x_{i}∼N_{p}(0,\Sigma )$, $\beta^{*}$ is the *p*-dimensional regression coefficient, $\epsilon_{i}∼N(0,\sigma_{y}^{2})$. We impose an orthogonal factor model for the covariates $x_{i}=u_{i}^{T}\Lambda_{d}V_{d}^{T}+e_{i},$ where *u_i_* are generated from $N_{d}(0,I_{d})$ independently, $\Lambda_{d}$ is a diagonal matrix with entries $\left(\lambda_{1},\ldots ,\lambda_{d}\right)$ in descending order, and $V_{d}\in R^{p\times d}$ with $V_{d}^{T}V_{d}=I_{d}$. The vector $e_{i}\in R^{n}$ has i.i.d. $N(0,\sigma_{x}^{2})$ entries independent of *u_i_*, and $\sigma_{x}>0$. We assume $V_{d}$ is row sparse with only *s* rows containing non-zero entries. These non-zero rows are the ‘true’ features to be discovered, and they correspond to $\beta^{*}\neq 0$.

It is important to note that, under this model, the rows of **X** follow a multivariate Gaussian distribution independently, with mean 0 and full-rank covariance $\Sigma =VLV^{T}$ whenever $\sigma_{x}^{2}>0$. Here, the columns of **V** are orthonormal eigenvectors on $R^{p}$ and the eigenvalues are $l_{1}\geq \cdots \geq l_{p}\geq 0$. Straightforward calculation shows that the first *d* columns in **V** are the same as the right singular vectors $V_{d}$ in the signal component of **X**. Furthermore, $l_{i}=\lambda_{i}^{2}1(i\leq d)+\sigma_{x}^{2},\;i=1,\ldots ,p$.

We generate $y\in R^{n}$ as a linear function of the latent factors $U_{d}$ with additive Gaussian noise: $y=U_{d}\Theta +z,$ where Θ is the regression coefficient, and *z_i_* are i.i.d. $N(0,\sigma_{y}^{2}),\;i=1,\ldots ,n$, independent of **X**. Under this model the population marginal correlation between each feature in **X** and *y* is $\Phi =V_{d}\Lambda_{d}\Theta ,$ and the population OLS coefficient of regressing *y* on **X** is $\beta^{*}=V_{d}L_{d}^{-1}\Lambda_{d}\Theta .$ Note that the number of non-zero $\beta^{*}$ is *s*, because $V_{d}$ has only *s* rows with non-zero entries.

In all cases, we use *n *=* *100 observations and *p *=* *1000 features, generating three equal-sized sets for training, validation and testing. We use prediction accuracy on the validation set to select tuning parameters for all methods. For the case of SuffPCR, this means only *λ*, because we choose *t* with Algorithm 2 and set *d *=* *3. We use the test set for evaluating out-of-sample performance. Each simulation is repeated 50 times. Results with *n *=* *200 and *p *=* *5000 were similar. Algorithm 3 makes this entire procedure more explicit.

We compare SuffPCR with a number of alternative methods. The Oracle estimator uses OLS on the true features and serves as a natural baseline: it uses information unavailable to the analyst (the true genes) but represents the best method were that information available. We also present results for Lasso (Tibshirani, 1996), Ridge (Hoerl and Kennard, 1970), Elastic Net (Zou and Hastie, 2005), SPC (Bair *et al.*, 2006), AIMER (Ding and McDonald, 2017), ISPCA (Piironen and Vehtari, 2018) and PCR using FPS directly without feature screening (using Algorithm 1 without Steps 9–11). For ISPCA, we use the dimreduce R package to estimate the principal components before performing regression. For all competitors, we choose any tuning parameters that do not have default values using the validation set. Examples are *λ* in Lasso, Ridge and Elastic Net or the initial thresholding step in SPC. We use the correct embedding dimension (*d *=* *3) whenever this is meaningful. Additional experiments are given in the Supplementary Material. There, we investigate conditions favorable to SPC, the choice of *d* and the impact of different SNR choices.

Algorithm 3 Generate synthetic data 1: **Input:** *n *=* *100, *p *=* *1000, *r *=* *5, *d *=* *3, ${\text{SNR}}_{x}={\text{SNR}}_{y}=5$.

2: Generate i.i.d. $N(0,1)\;U\in R^{n\times d}$, $E\in R^{n\times p},\;z\in R^{n}$.

3: Set $\Lambda_{d}=\text{diag}((d,\;d-1,\ldots ,1))\in R^{d\times d}$.

4: Generate i.i.d. $N(0,1)\;\tilde{V}\in R^{d\times d}$ and orthogonalize the columns.

5: Extend $\tilde{V}\in R^{s\times d}$ by repeating each row *r* times (*s* = *rd*).

6: Set $V_{d}^{T}=[{\tilde{V}}^{T}\;0]\in R^{d\times p}$.

7: Generate i.i.d. $N(0,1)\;\tilde{\Theta }\in R^{d-1}$.

8: Set $\Theta_{d}=-\left(\sum_{i=1}^{d-1}{\tilde{V}}_{ri}^{T}\Lambda_{ii}{\tilde{\Theta }}_{i}\right)/({\tilde{V}}_{rd}\Lambda_{dd})$.

9: Set $\Theta ={\left[{\tilde{\Theta }}^{T}\;\Theta_{d}\right]}^{T}$.

10: Set $\beta^{*}=V_{d}L_{d}^{-1}\Lambda_{d}\Theta$.

11: Set $\sigma_{x}^{2}=\text{tr}(\Lambda_{d}^{2})/(p{\text{SNR}}_{x}^{2})$

12: Set $\sigma_{y}^{2}=\left(\beta^{*T}V_{d}^{T}\Lambda_{d}^{2}V_{d}\beta^{*}+\sigma_{x}^{2}||\beta^{*}|{|}_{2}^{2}\right)/(n{\text{SNR}}_{y}^{2})$.

13: Set $X=U_{d}\Lambda_{d}V_{d}^{T}+\sigma_{x}E$ and $y=U_{d}\Theta +\sigma_{y}z$

#### 2.2.2 Conditions favorable to SuffPCR

The first setting is designed to show the advantages of SuffPCR relative to alternative methods, especially SPC. We note that other methods that employ screening by the marginal correlation (Ding and McDonald, 2017; Piironen and Vehtari, 2018) will have similar deficiencies. Because SPC works well if Equation (1) holds, we design Σ to violate this condition and set the first 15 features to have non-zero $\beta^{*}$ but allow only the first 10 features to have non-zero correlation with the phenotype. This behaviour is achieved with Line 8 of Algorithm 3. By solving this equation in one unknown component of Θ, we force Φ = 0 for the third group of 5 components. Thus, as described in above, Equation (1) will not hold: some $\Phi_{j}=0$ but $\beta_{j}^{*}\neq 0$. We set the true dimension of the subspace as *d *=* *3, and we use the correct dimension for methods based on principal components.

**Fig. 2.**
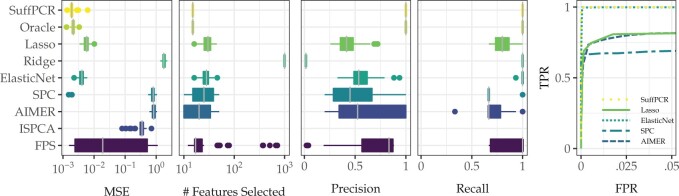
This figure compares the performance of SuffPCR against alternatives when the features come from a row-sparse factor model under favorable conditions for SuffPCR. Boxplots and ROC curve (far right figure) are over 50 replications. We have omitted the other methods from the ROC curve for legibility, but their behavior is similar to lasso. TPR and FPR stand for true/false positive rate, respectively. Note that (as one would expect from the simulation conditions) SPC has the worst performance in terms of the ROC curve while both SuffPCR and Elastic net have AUC of almost 1

Figure 2 shows the performance of SuffPCR and state-of-the-art alternatives. In addition to reporting each method’s prediction MSE on the test set, we also show the number of features selected, precision, recall and the receiver operating characteristic (ROC) curve. The ISPCA implementation does not select features. In this example, SuffPCR actually outperforms the oracle estimator, attaining smaller MSE while generally selecting the correct features. This seemingly implausible result is likely because the variance of estimating OLS on 15 features is large relative to that of estimating the low-dimensional manifold followed by 3 regression coefficients. SuffPCR has a clear advantage over all the alternative methods, especially SPC which is three orders of magnitude worse. SPC works so poorly because it ignores five features. ISPCA has slightly lower MSE than SPC. Ridge is the worst, due to fitting a dense model when a sparse model generated the data. SuffPCR reduces MSE significantly relative to simply using FPS due to more accurate feature selection. The right plot in Figure 2 further shows the ROC curve for SuffPCR, Lasso, Elastic Net, SPC and AIMER in which we can easily vary the tuning parameter and select various numbers of features. SuffPCR and AIMER have a perfect ROC curve, while the other three methods are unable to identify five features. We undertake a similar exercise under conditions favorable to SPC in the Supplementary Material.

#### 2.2.3 Semi-synthetic analysis with real genomics data

**Fig. 3.**
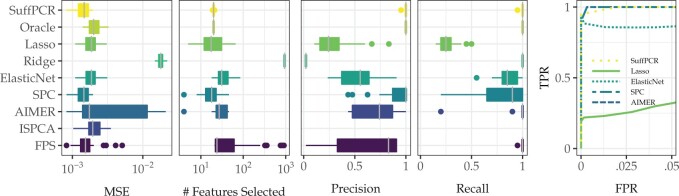
This figure compares the performance of SuffPCR against alternatives when the features come from a row-sparse factor model extracted from the NSCLC data. Boxplots and ROC curve (far right figure) are over 50 replications. In terms of the ROC curve, SPC and AIMER have the best performance, though SuffPCR is not far behind. But note that SPC has much worse precision and recall

The simulations in Section 2.2.2 explore various scenarios for the data generation process and show the performance of SuffPCR relative to the alternatives; however, they do not use any real genomic data. In this section, rather than fully generating **X**, we create a semi-synthetic analysis wherein only the phenotypes are generated. We first performed PCA on the NSCLC data (Lazar *et al.*, 2013) and note that the first two empirical eigenvalues are relatively large, so we chose the number of PCs to be *d *=* *2. We keep the top 20 rows in the empirical **V** which have the largest norm and set the rest to 0. We then recombine and add noise. The phenotype is constructed as in the previous simulations, and the SNR is calibrated as above. Figure 3 shows the results analogous to those in Figure 2. SuffPCR continues to perform well relative to alternatives, though here, FPS has similar MSE, albeit poor feature selection.

### 2.3 Analysis of real genomics data

We analyze five microarray datasets that are publicly available and widely used as benchmarks. Four of the datasets present mRNA abundance measurements from patients with breast cancer (Van’t Veer *et al.*, 2002; Miller *et al.*, 2005), diffuse large B-cell lymphoma (DLBCL) (Rosenwald *et al.*, 2002) and AML (Bullinger *et al.*, 2004), and the fifth reports microRNA (miRNA) levels from NSCLC patients (Lazar *et al.*, 2013). The features in **X** are gene expression measurements from microarrays. In the Supplementary Material, we apply SuffPCR to predict COVID-19 viral load from RNA-Seq data.

The phenotypes *Y* are censored survival time in all cases, though some of the datasets also contain binary survival status indicators. Because the real valued phenotype is non-negative and right censored, we follow common practice and transform *Y* to log(Y+1). Each observation is a unique patient. The first breast cancer dataset has 78 observations and 4751 genes, the second has 253 observations and 11 331 genes, DLBCL has 240 observations and 7399 genes, AML has 116 observations and 6283 genes and NSCLC has 123 observations and 939 genes.

We randomly split each dataset into 3-fold for training, validation and testing with proportions 40%, 30% and 30% respectively. We set the number of components *d *=* *3 and search over 5 log-linearly spaced *λ* values. Other choices for *d* and *λ* yield similar results. We train all methods on the training set, use the validation set to choose any necessary tuning parameters and report performance of each method on the test set. We repeat the entire process (data splitting, validation and testing) 10 times to reduce any bias induced by the random splits. In all cases, all methods were tuned to optimize validation-set MSE.

Table 2 shows the average prediction MSE and the average number of selected features for SuffPCR and any alternative methods that perform feature selection. SuffPCR works better than all the alternative methods on 4 out of 5 datasets with a comparatively small number of features selected. The DLBCL data are difficult for both sparse and PC-based methods. As described above, FPS cannot be used for these data sets because of the number of genes. Non-sparse alternatives have much smaller MSE, suggesting that many genes may play a roll in mortality rather than only a subset. SPCA is designed to maximize the variance explained by the principal components subject to a penalty on the non-sparsity, and it does not seem to work well in regression tasks. DSPCA has relatively low prediction MSE, and it does in principle perform feature selection, though it generally produces a dense model. While Ridge, Random Forests and SVM predict well in general, they do not perform any feature selection, which is a key objective here, so show their MSE in the Supplementary Material.

**Table 2.** Prediction MSE and number of selected features for regression of survival time on gene expression measurements

	Breast Cancer1		Breast Cancer2		DLBCL		AML		NSCLC	
Method	MSE	Feature #	MSE	Feature #	MSE	Feature #	MSE	Feature #	MSE	Feature #
SuffPCR	**0.5980**	80	**0.4168**	121	0.7073	48	**1.9568**	75	**0.1970**	27
Lasso	0.7141	7	0.4622	39	0.6992	31	2.0998	3	0.2263	4
ElasticNet	0.6845	41	0.4517	104	**0.6869**	87	2.0820	5	0.2332	20
SPC	0.6188	59	0.4179	823	0.7677	67	2.3237	62	0.2795	62
ISPCA	0.8647	NA	0.5882	NA	0.9441	NA	2.3109	NA	0.2408	NA
AIMER	0.6629	76	0.4192	795	0.7003	76	1.9737	36	0.2120	50
SPCA	17.0965	212	4.7239	38	2.5980	652	31.11	1043	0.9757	387
DSPCA	0.6132	4374	0.4557	7880	0.7249	1342	1.9781	2742	0.2041	305

Bolded text emphasizes the method with the lowest MSE.

To assess the potential relevance of the genes selected by SuffPCR to the cancer type from which they were identified, we further explored the DLBCL data and extracted the selected genes. (We do the same with AML in the Supplementary Material.) We first find the best *λ* via 5-fold cross-validation on all the data and then train SuffPCR with this *λ*. Our model selects 87 features corresponding to 32 unique genes and 2 expressed sequence tags (ESTs) for DLBCL. Seventeen of the identified genes encode ribosomal proteins, overexpression of which is associated with poor prognosis (Ednersson *et al.*, 2018). A further nine genes encoding major histocompatibility complex class II (MHCII) proteins were detected, a notable finding in light of the fact that MHCII downregulation is a means by which some DLBCLs evade the immune system (de Charette and Houot, 2018). Discovering these large groups of similarly functioning genes illustrates the benefits of SuffPCR relative to alternatives. *CORO1A* encodes the actin-binding tumor suppressor p57/coronin-1a, the promoter of which is often hypermethylated, and therefore likely silenced in DLBCL (Li *et al.*, 2002). *FEZ1* expression has been used in a prognostic model (Liu *et al.*, 2019). *RAG1*, encoding a protein involved in generating antibody diversity, can induce specific genetic aberrations found in DLBCL (Miao *et al.*, 2019). *RYK* encodes a catalytically dead receptor tyrosine kinase involved in Wnt signaling and *CXCL5* encodes a chemokine. To our knowledge, neither gene has been implicated in DLBCL and thus may be of interest for further exploration. EST Hs.22635 (GenBank accession AA262469) corresponds to a portion of *ZBTB44*, which encodes an uncharacterized transcriptional repressor, while EST Hs.343870 (GenBank accession AA804270) does not appear to be contained within an annotated gene. The Supplementary Material lists the selected genes and associated references. A separate listing of the genes encoding ribosomal and MHCII proteins are given in the Supplementary Material.

### 2.4 Theoretical guarantees

When the sparse factor model described in Section 2.2.1 is true, SuffPCR enjoys near-optimal convergence rates. We now make the necessary assumptions concrete and note that some can be weakened.

A1 $Y_{i}=X_{i}^{T}\beta^{*}+\epsilon_{i},\;i=1,\ldots ,n$, where $\epsilon_{i}∼N(0,\sigma_{y}),\;\sigma_{y}>0$.

A2 $X_{i}∼N_{p}(0,\Sigma ),\;i=1,\ldots ,n$.

A3 $\Sigma =VLV^{T}$, is symmetric, $V^{T}V=I_{p}$, **L** is diagonal.

A4 $l_{i}=\lambda_{i}^{2}1(i\leq d)+\sigma_{x}^{2}$ and $\lambda_{1}-\lambda_{d}:=\phi >0$.

A5 $||\text{diag}\left(V_{d}V_{d}^{T}\right)|{|}_{0}\leq s$ and $\min_{j}\left\{{\left(V_{d}V_{d}^{T}\right)}_{jj}\vee 0\right\}>2\tau$.

A6 as $n,p\to \infty ,\;n>(s^{2}+d)\;\text{log}(p)$ eventually.

Assumptions A1–A4 are the same as those used in Section 2.2.1 to generate data from a linear factor model. Assumption A5 says that the number of true nonzero coefficients $\beta^{*}$ must be no more than *s* and that the size of the associated components must be large enough. Assumption A6 means that eventually, we must have at least as many observations *n* as a logarithmic function of *p* times the true number of components plus the square of the number of nonzero $\beta^{*}$ coefficients.

Theorem 1. *Suppose Assumptions A1–A6 hold and let* $\hat{\beta }$*be the estimate produced by* SuffPCR *with* $\lambda =c\lambda_{1}\sqrt{\;\text{log}(p)/n}$*and* t<2τ*where t is the threshold used in Algorithm 1 and τ is given in A5. Then*

$$\frac{1}{n}||X(\hat{\beta }-\beta^{*})|{|}_{2}^{2}=O_{P}\left(\frac{\left(s^{2}+d)\;\text{log}(p\right)}{n}\right).$$

Theorem 2. *Suppose Assumptions A1–A6 hold and let* $\hat{\beta }$*be the estimate produced by* SuffPCR *with* $\lambda =c\lambda_{1}\sqrt{\;\text{log}(p)/n}$*and* 2τ>t>τ*where t is the threshold used in Algorithm 1 and τ is given in A5. Then*

$$\left|\text{supp}\left(\hat{\beta }\right)△\text{supp}\left(\beta^{*}\right)\right|=O_{P}\left(\frac{s^{2}\;\text{log}(p)}{n}\right),$$

*where* A△B=A/B∪B/A*is the symmetric difference operator and* supp*denotes the support set.*

In both results above, *c* is a positive number (possibly different between the two) that is independent of *n* and *p* but may depend on any of the other values given in A1–A6. Theorem 1 gives a convergence rate for the prediction error of SuffPCR comparable to that of Lasso though with explicit additional dependence on *d*. Under standard assumptions with fixed design, this dependence would not exist for Lasso. On the other hand, our results are for random design with *d* small, along with different constants absorbed by the big-O. Theorem 2 shows that our procedure can correctly recover the set of nonzero $\beta^{*}$ as long as the threshold *t* is chosen correctly. We note that this result is a direct consequence of Vu *et al.* (2013, Theorem 3.2). In practice, the condition 2τ>t>τ cannot be verified, although the ‘elbow’ condition we employ in the empirical examples seems to work well. Finally, we emphasize that, as is standard in the literature, these results are for asymptotically optimal tuning parameters λ, t rather than empirically chosen values. The proof of Theorem 1 is given in the Supplementary Material. These results suggest that SuffPCR is nearly optimal as *p* and *n* grow.

## 3 Discussion

High-dimensional prediction methods, including regression and classification, are widely used to gain biological insights from large datasets. Three main goals in this setting are accurate prediction, feature selection and computational tractability. We propose a new method called SuffPCR which is capable of achieving these goals simultaneously. SuffPCR is a linear predictor on estimated sparse principal components. Because of the sparsity of the projected subspace, SuffPCR usually selects a small number of features. We conduct a series of synthetic, semi-synthetic and real data analyses to demonstrate the performance of SuffPCR and compare it with existing techniques. We also prove near-optimal convergence rates of SuffPCR under sparse assumptions. SuffPCR works better than alternative methods when the true model only involves a subset of features.

## Funding

The authors gratefully acknowledge support National Science Foundation (grant DMS–1753171 to D.J.M.), the National Institutes of Health (grant R35GM128631 to G.E.Z.) and the National Sciences and Engineering Research Council of Canada (NSERC) (grant RGPIN-2021-02618 to D.J.M.).

*Conflict of Interest*: none declared.

## Supplementary Material

vbac033_Supplementary_Data
