# Supplementary material for: Sufficient principal component regression for pattern discovery in transcriptomic data
Source: Bioinform Adv. 2022 May 14;2(1):vbac033. doi: 10.1093/bioadv/vbac033 (PMC9194947; doi:10.1093/bioadv/vbac033)
Supplement: vbac033_Supplementary_Data [file vbac033_supplementary_data.pdf]

Supplement for: Sufficient principal component regression for pattern discovery in transcriptomic data

L. Ding, G.E. Zentner, and D.J. McDonald

1 Genetics data discussion

In this section we provide more details on the 5 standard genetics data sets discussed in the manuscript. We then apply our methodology to one/two additional data sets which are less well studied in the literature.

1.1 Detailed description

In the manuscript (and below in Section 3.4), we analyze 5 microarray datasets that are publicly available and widely used as benchmarks. Four of the datasets present messenger RNA (mRNA) abundance measurements from patients with breast cancer (Van't Veer et al., 2002; Miller et al., 2005), diffuse large B-cell lymphoma (DLBCL) (Rosenwald et al., 2002), and acute myeloid leukemia (AML) (Bullinger et al., 2004), and the fifth reports microRNA (miRNA) levels from non-small cell lung cancer (NSCLC) patients (Lazar et al., 2013).

These data sets have between about 80 and 250 patients with expression measurements on 900 to 11000 genes. Table 1 gives specific statistics about each dataset.

Name	n (patients)	p (genes)
Breast cancer (Van't Veer et al., 2002)	78	4751
Breast cancer (Miller et al., 2005)	253	11331
DLBCL (Rosenwald et al., 2002)	240	7399
AML (Bullinger et al., 2004)	116	6283
NSCLC (Lazar et al., 2013)	123	939

Table 1. Summary of canonical datasets

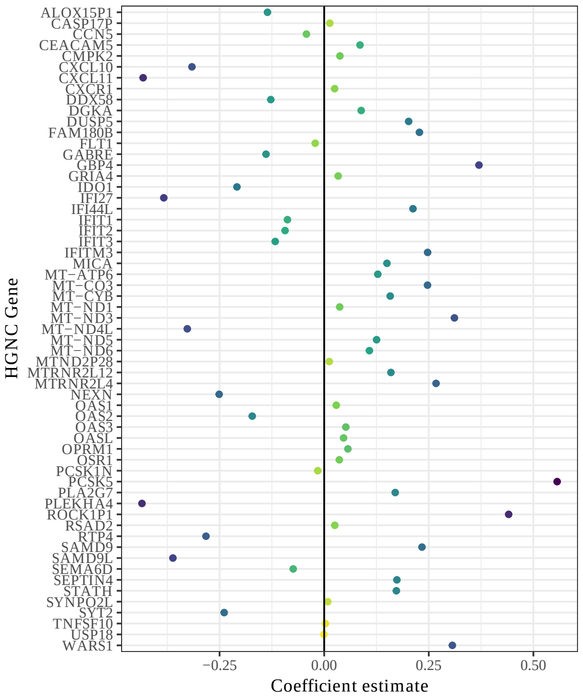

Fig. 1. Coefficient estimates from `SuffPCR` on RNA-Seq measurements of COVID-19-positive patients. The colour corresponds to the magnitude.

The AML data was originally collected and analyzed by (Bullinger et al., 2004). They used complementary-DNA microarrays to measure gene expression from either peripheral-blood or bone marrow samples from 116 adults with AML. The gene expression measurements for the 6283 genes that were highly variable across patients are included. The observed outcomes are the (possibly right-censored) survival time in days as well as a binary indicator for whether or not the patient died.

The first set of breast cancer data from (Van't Veer et al., 2002) is based on 78 sporadic lymph-node-negative patients. The authors derived cRNA from snap-frozen tumor samples and pooled across each of the sporadic carcinomas. The 4751 available genes were significantly regulated across the patients (showing at least a two-fold difference across at least 5 patients). The outcome is the follow-up time (in months) for metastases as well as a binary indicator for whether prognosis was "good" (no metastases within 5 years) or "poor".

The second set of breast cancer gene expression measurements is based on 253 patients who's cancer tissues were sequenced for the p53 mutation (Miller et al., 2005). A binary variable for the presence of the p53 mutation is missing for two patients, which were removed from further analysis. The regression exercise focuses on the right-censored survival time (measured in years). The measured gene expressions come from Affymetrix high-density oligonucleotide arrays. A number of other clinical variables are also present in the data contained in the R package (`https://github.com/dajmcdon/suffpcr`).

The DLBCL data was collected by (Bullinger et al., 2004) and contains Biopsy samples from 240 patients with gene expression measurements from DNA microarrays. The microarrays were constructed from 12,196 clones of cDNA then used to quantify the expression of mRNA in the tumors. Genes with significant differential expression across patients were included. The response is survival time after chemotherapy in years as well as binary survival status (57% of patients died).

Finally, the NSCLC dataset was analysed by (Lazar et al., 2013). The expression measurements consist of from paired (tumor and non-tumor) micro RNA collected from 123 cancer patients. The response variable is the time to relapse in years. Other clinical variables are also available.

2 Application to COVID-19 Data

In this section, we apply our methods to RNA-Seq data for COVID-19-positive patients. As a developing disease, biological analysis remains highly desirable, and so to facilitate this sort of undertaking, recent work has endeavoured to collect and distribute this data publicly (Zhang et al., 2021).

Here, we examine the data collected by Lieberman et al. (2020), and examine how well RNA-Seq measurements predict viral load. The data contains results for 430 PCR-confirmed COVID-positive patients and 54 negative controls. Lieberman et al. (2020) examines both differential expression between positives and controls and the viral load of the positive patients, while we focus on only the viral load. Of the 430 confirmed positives, 413 have reported usable measurements. Viral load in this case is measured through a proxy: the cycle threshold (Ct) of the SARS-CoV-2

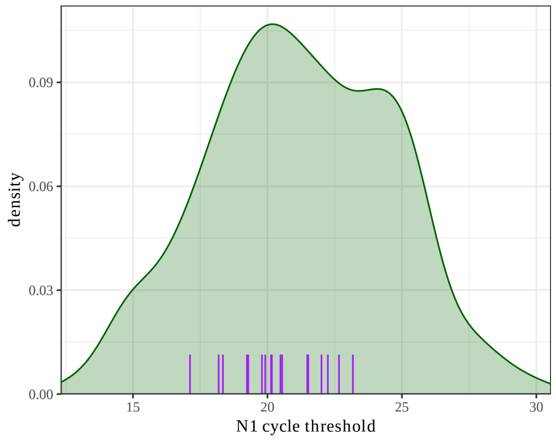

Fig. 2. Density of observed N1 Ct values for 413 patients (green area) and predicted values for 27 positive patients with "Unknown" values (purple lines) based on `SuffPCR` estimated from the complete cases.

nucleocapsid gene region 1 (N1) target from a PCR test. Larger Ct values indicate more cycles are required to detect the N1 target, and thus, likely indicate *lower* viral load. While Lieberman et al. (2020) bin this continuous measurement into "high" and "low" groups to undertake differential analysis, our methodology directly models the continuous response. This allows for (1) increased ability to detect potentially predictive genes and (2) direct quantification of the effect of increased expression on viral load.

The raw expression measurements are preprocessed before being used in `SuffPCR`. First, we remove any genes whose median expression level across the 413 patients with Ct measurements is 0. This reduces the data from ~36,000 genes to 9435. We then transform the raw counts using $\tilde{z} \mapsto \log(\tilde{z}+1)$. Finally, we centre and scale by the mean and standard deviation of the similarly transformed measurements from the healthy controls. Mathematically, if $\tilde{z}_{gt}$ is the vector of expression measurements for gene g in the treatment group and $\tilde{z}_{gc}$ is the vector of expression measurements for the same gene in the control group, we form

$$x_{gt} = \frac{\log(\tilde{z}_{gt}+1) - \overline{z}_{gc}}{\mathrm{sd}(z_{gc})},$$

where $\overline{z}_{gc} = \mathrm{mean}(\log(\tilde{z}_{gc}+1))$ and $\mathrm{sd}(z_{gc}) = \mathrm{sd}(\log(\tilde{z}_{gc}+1))$.

We apply `SuffPCR` to the $\mathbf{X}$ matrix formed by the columnwise concatenation of x_{gt} with the N1 Ct as the response vector. We examined embedding sizes of $d \in \{3,\ 5,\ 15\}$ and λ on a $\log_{10}$-spaced grid between 0 and 1. We chose both parameters using the minimum of the 5-fold cross-validation error. The result is shown in Figure 1 (here $d = 15$). Our method selected 59 genes. Many of these with the largest magnitude (darkest colour) are similar to those described in (Lieberman et al., 2020): the CXCL10 and 11 genes along with IDO1 and IFI27 are proinflammatory and/or interferon induced and may be related to the "cytokine storm" found in some patients (Lorè et al., 2021). Novel and potentially interesting PLEKHA4, which is strongly related to higher viral load, but more closely related to melanomas. PCS5K is more strongly expressed in patients with lower viral load. This gene encodes a proprotein convertase that may potentially help to clear the virus. ROCK1P1, also more strongly expressed in lower viral load patients, is not well understood.

As mentioned above, 27 patients were positive but did not have a measured N1 Ct from the PCR test. These were also missing age and gender information. Using the fitted `SuffPCR` model, we can predict their missing N1 cycle thresholds. These predictions are shown in Figure 2. The green area shows a density estimate for the 413 patients with observed measurements while the purple lines display predicted values for those positive patients whose N1 Ct values are labeled "Unknown". Because the data is missing, the accuracy of these predictions cannot be determined.

3 Additional experiments and results for regression

3.1 Conditions favorable to SPC

This example is designed to show the performance of `SuffPCR` under favorable conditions for SPC. Here, the only alteration to the data generation process is that we set the first 10 features to have non-zero β^*, the same features which have non-zero marginal correlations with the phenotype. This is achieved using Algorithm 3 in the manuscript with a few alterations: (1) in line 5, we generate $\widetilde{\mathbf{V}}_d \in \mathbb{R}^{(d-1)\times 3}$ (recall that we have chosen $d = 3$); and (2) we replace lines 7–9 by directly simulating $\Theta \in \mathbb{R}^d$ with i.i.d. standard normal entries.

Figure 3 gives the analogous results for this example. Here, `SuffPCR` has comparable MSE to SPC, which is the best, slightly better than the oracle as was the case for `SuffPCR` above. However, SPC is less likely to select the correct features, while `SuffPCR` selects the correct features most of the time. Furthermore, `SuffPCR` tends to select fewer features, and it has the best precision and recall (Ridge will always have recall equal to 1).

3.2 Selecting tuning parameters

In `SuffPCR`, the tuning parameters are the penalty term λ, the dimension of the projected subspace d, and the threshold t. In all the simulations, we examine the same values of λ. When analyzing real datasets, our algorithm offers automatic calculation of potential values of λ given the sample covariance matrix. In practice, the more penalty parameters we explore, the better the results could be assuming the sample size is large enough that risk estimation is not too volatile.

To demonstrate the importance of selecting d, we simulate the data with $d = 3$ and set all the other parameters the same as in the first simulation. We estimate `SuffPCR` using $d \in \{1,\ 3,\ 5\}$ and display the results in Figure 4. When d is smaller than the true value, it is hard for `SuffPCR` to capture all the information contained in $\mathbf{X}$, thus `SuffPCR` has terrible prediction MSE, worse than any other methods. When d is larger than the true value, `SuffPCR` remains reasonable because its bias is small, but the variance will eventually increase as d increases, diminishing performance. Selecting t is not difficult in our simulations, so the results have been omitted, but it is less trivial in the real data settings.

3.3 Investigation of signal-to-noise ratio with synthetic data

In all or our synthetic examples, the data generating model has two sources of noise, one from constructing $\mathbf{X}$ corresponding to σ_x, and the other from constructing Y, denoted σ_y. This simulation aims to control the two noise sources separately to examine their effect on the performance of `SuffPCR`. Note that $\mathbf{X}$ is random unlike in standard prediction studies, so both sources of noise are important. We use SNR_x and SNR_y to denote the signal-to-noise ratio for $\mathbf{X}$ and Y respectively. These are given by

$$\mathrm{SNR}_x = \frac{\mathbb{E}\left[\left\|\mathbf{U}_d\mathbf{\Lambda}_d\mathbf{V}_d^{\mathsf{T}}\right\|_F\right]}{\mathbb{E}\left[\|\sigma_x\mathbf{E}\|_F\right]} = \sqrt{\frac{\mathrm{tr}(\Lambda_d^2)}{p\sigma_x^2}},$$

$$\mathrm{SNR}_y = \frac{\mathbb{E}\left[\|\mathbf{X}\beta^*\|_2\right]}{\mathbb{E}\left[\|\sigma_y Z\|_2\right]} = \sqrt{\frac{\beta^{*\mathsf{T}}\mathbf{V}_d\mathbf{\Lambda}_\mathbf{d}^2\mathbf{V}_\mathbf{d}^{\mathsf{T}}\beta^* + \sigma_x^2\|\beta^*\|_2^2}{n\sigma_y^2}}.$$

Note that SNR_y depends not only on β^* but also on σ_x and the linear manifold through $\mathbf{V}_d$.

We alter the values of SNR_x and SNR_y to generate $\mathbf{X}$ and Y while everything else is as in the first simulation. Figure 5 shows the prediction

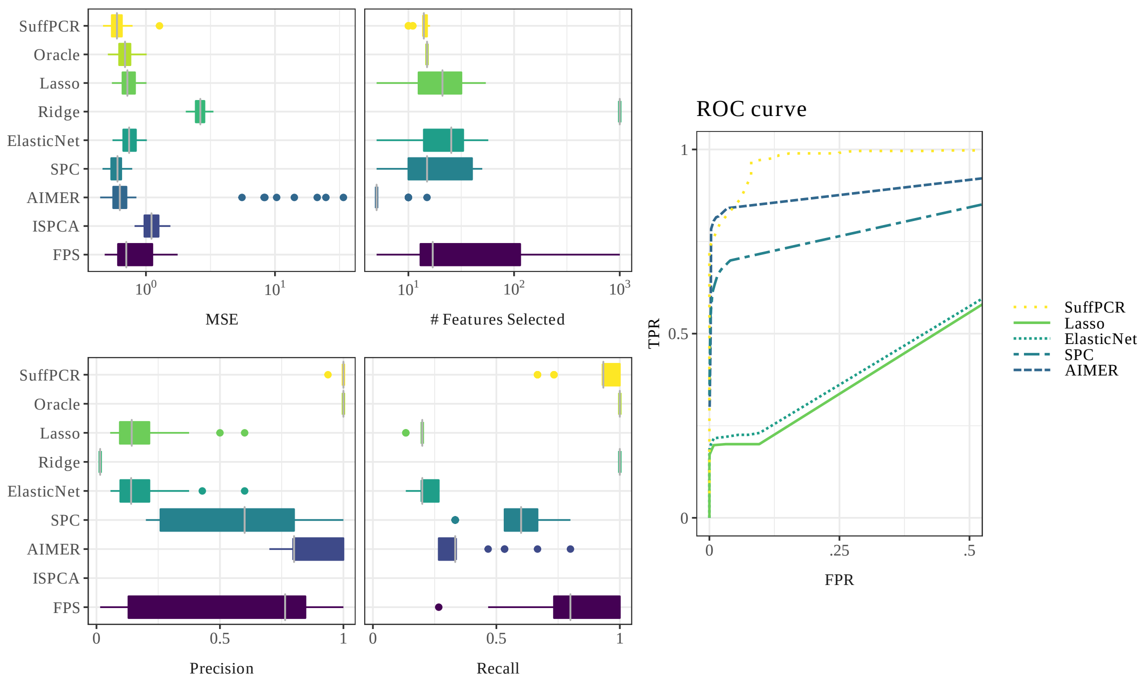

Fig. 3. This figure demonstrates the performance of `SuffPCR` under favorable conditions for SPC. We have omitted the other methods from the ROC curve for legibility, but their behavior is similar to lasso.

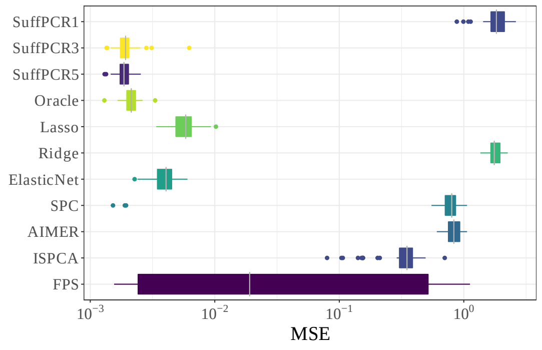

Fig. 4. This figure compares the prediction MSE of `SuffPCR` with alternatives with different d in `SuffPCR`.

Gene	Related ?	Reference
Ribosomal protein genes (17)	✔	Ednersson et al. (2018)
MHCII (9)	✔	de Charette and Houot (2018)
CORO1A	✔	Li et al. (2002)
FEZ1	✔	Liu et al. (2019)
RAG1	✔	Miao et al. (2019)
RYK	✗	
CXCL5	✗	
ESTs Hs.22635, Hs.343870	✗	

Table 2. Predictive genes for DLBCL selected by `SuffPCR`.

MSE for all methods on four sets of simulated data for both high and moderate combinations of SNR_x and SNR_y. When the SNR decreases, the prediction MSE of all the methods increases as expected. In all configurations, `SuffPCR` performs similarly to the oracle, better than Lasso or Elastic Net, while SPC is more similar to Ridge regression (though better). Interestingly, changing SNR_x and SNR_y has a similar impact on nearly all the methods except Ridge, SPC, AIMER, and ISPCA, which are nearly unaffected and uniformly worse by an order of magnitude.

3.4 Predictive genes/miRNA for additional cancers

Table 2 enumerates genes selected by `SuffPCR` on the DLBCL data, Table 3 lists the selected genes for AML, Table 4 lists the miRNA sequences selected by `SuffPCR` on the NSCLC data, while Table 5 lists all the features selected by `SuffPCR` on the Breast Cancer1 data. Table 6 gives the prediction MSE for Ridge, Random Forests and SVM for the 5 regression datasets we examine in Section 3.3 of the manuscript. These methods predict well in general, but they do not select features.

For AML we describe the related work summarized in Table 3 in more detail. Of the 4 discovered genes, 3 have been discussed in the literature. *BPGM*, encoding a multifunctional metabolic enzyme restricted to erythrocytes and placental cells, is upregulated in mouse models of AML (Novak et al., 2012). *PI4KB*, encoding a lipid kinase, is amplified in breast cancer (Waugh, 2014) and has been proposed to be a druggable AML-specific dependency (Zhou et al., 2020). *LOC90379* encodes an uncharacterized protein that is highly immunogenic in ovarian cancer serum (Gnjatic et al., 2010). The fourth feature discovered, Hs.321434, is an EST (GenBank accession H96229) that corresponds to an intronic sequence of an uncharacterized long noncoding RNA (lncRNA), LOC101929579, and is thus of uncertain significance. Similar listings for the discovered genes for the NSCLC and Breast Cancer 1 data are given in the Supplement without the accompanying literature review.

Finally, Table 8 expands on the listing in the main document, listing the genes encoding ribosomal proteins and MHCII protein which predict DLBCL survival as selected by `SuffPCR`.

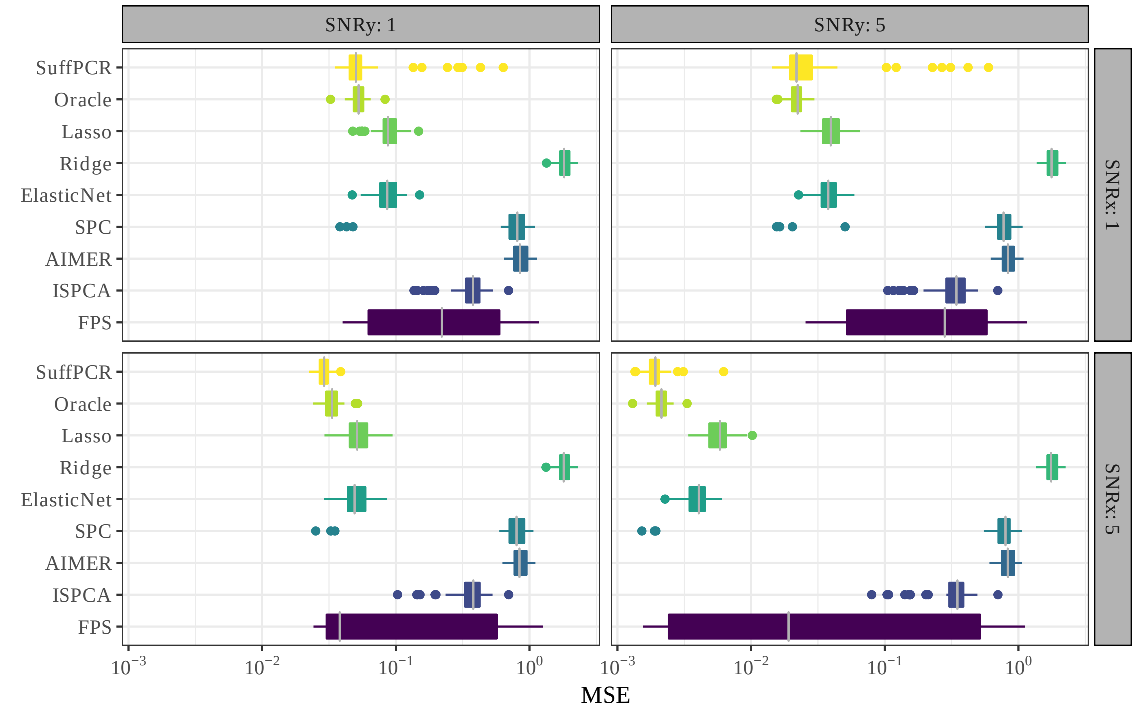

Fig. 5. This figure compares the prediction MSE of `SuffPCR` with alternatives across different values of SNR_x and SNR_y. The x-axis is on the log scale. Boxplots are over 50 replications.

Gene	Related ?	Reference
BPGM	✔	Novak et al. (2012)
PI4KB	✔	Zhou et al. (2020)
EST Hs.321434	✗	
LOC90379	✔	Gnjatic et al. (2010)

Table 3. Predictive genes for AML selected by `SuffPCR`.

4 Extension to classification

`SuffPCR` is easily extended to solve classification tasks using logistic regression (or even other classification methods). We use logistic regression as an example to show how `SuffPCR` performs for classification tasks. Note that the algorithm is similar to Algorithm 1 except that step 12 is replaced by the objective of logistic regression.

4.1 Synthetic data for binary classification

We first use a simulation to demonstrate the generalization of `SuffPCR` to solve a binary classification problem. Using the same data generating model and parameters as in the favorable scenario in Section 3.1.1 of the manuscript, we generate Y using the logistic function. As before, we choose tuning parameters with the validation set, and report the overall prediction accuracy on the test set.

Figure 6 shows the classification accuracy compared to the oracle (logistic regression on the true predictors), logistic lasso, logistic ridge, and logistic FPS (logistic regression on the estimated principal components from FPS). `SuffPCR` has very high classification accuracy on the test set relative to the alternative methods. We also simulate classification data analogously to the other scenarios discussed in the manuscript, and the results are very similar.

#	Sequence	#	Sequence
1	hsa-miR-376c	21	hsa-miR-409-5p
2	hsa-miR-320c	22	hcmv-miR-US4
3	hsa-miR-299-3p	23	hsa-miR-376a
4	hsa-miR-154	24	hsa-miR-1471
5	hsa-miR-410	25	hsa-miR-411
6	hsa-miR-1182	26	bkv-miR-B1-5p
7	hsa-miR-136	27	hsa-miR-377
8	hsa-miR-379	28	hsa-miR-601
9	hsa-miR-765	29	hsa-miR-299-5p
10	hsa-miR-610	30	hsa-miR-543
11	hsa-miR-487a	31	hsa-miR-381
12	hsa-miR-136*	32	hsa-miR-329
13	hsv1-miR-H1	33	hsa-miR-760
14	hsa-miR-622	34	hsa-miR-409-3p
15	hsa-miR-659	35	hsa-miR-617
16	hsa-miR-376b	36	hsa-miR-758
17	hsa-miR-154*	37	hsa-miR-1183
18	hsa-miR-483-5p	38	hsa-miR-671-5p
19	hsa-miR-337-5p	39	hsa-miR-127-3p
20	hsa-miR-376a*		

Table 4. RNA sequences selected by `SuffPCR` for NSCLC data.

4.2 Analysis of real genomics data

We analyze 3 of the 5 real genomics datasets from Section 3.3 of the manuscript, which include a binary survival status. We use the same training test split process as in the manuscript and compare `SuffPCR` with logistic lasso and logistic ridge.

Table 7 shows the average classification accuracy and average number of selected genes in the classification tasks. The results in the classification

#	Feature	#	Feature
1	NM_003118	15	NM_003247
2	Contig46244_RC	16	NM_004079
3	Contig55801_RC	17	NM_002775
4	M37033	18	NM_004369
5	NM_004385	19	NM_002985
6	Contig43613_RC	20	Contig43833_RC
7	Contig42919_RC	21	NM_005565
8	NM_016081	22	Contig52398_RC
9	NM_016187	23	NM_006889
10	Contig30260_RC	24	Contig66347
11	NM_000089	25	NM_000090
12	NM_000138	26	Contig25362_RC
13	NM_000393	27	NM_000560
14	Contig58512_RC	28	NM_001387

Table 5. Features selected by `SuffPCR` for Breast Cancer1 data.

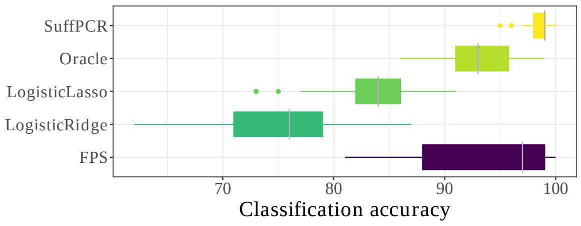

Fig. 6. This figure compares the classification accuracy of `SuffPCR` with alternative methods. The x-axis shows the percentage of correct classifications on the test set. Boxplots are over 50 repeated simulations.

tasks are similar to those in regression. Again, the DLBCL dataset tends to be difficult for both sparse and PC-based methods.

5 Approximate singular value decomposition in Algorithm 1

To approximate the top j eigenvalues of a symmetric matrix $\mathbf{A}$, we require the k-step (partial) Lanczos bidiagonalization ($j < k$). For initial vector p_1, this is given by

$$\mathbf{A}\mathbf{P}^{(k)} = \mathbf{Z}^{(k)}\mathbf{W}^{(k)}, \ \mathbf{A}^\mathsf{T}\mathbf{Z}^{(k)} = \mathbf{P}^{(k)}\mathbf{W}^{(k),\mathsf{T}} + r^{(k)}e_k^\mathsf{T}$$

where $\mathbf{P}^{(k),\mathsf{T}}\mathbf{P}^{(k)} = \mathbf{Z}^{(k),\mathsf{T}}\mathbf{Z}^{(k)} = \mathbf{I} \in \mathbb{R}^{k\times k}$, $\mathbf{P}^{(k),\mathsf{T}}r^{(k)} = 0$, $\mathbf{P}^{(k)}e_1 = p_1$, and $\mathbf{W}^{(k)}$ is bidiagonal. Approximate eigenvectors and eigenvalues for $\mathbf{A}$ can then be computed using the SVD for $\mathbf{W}$, which is easy because of the bidiagonal structure. However, the accuracy is closely tied to the choice of the initial vector p_1 and can be measured by the norm of the residual vector $r^{(k)}$. AIRLB (Baglama and Reichel, 2005) essentially augments the SVD of $\mathbf{W}$ with additional information to reexpress $\mathbf{P}^{(k)}$, $\mathbf{Z}^{(k)}$ and $\mathbf{W}^{(k)}$. This process is repeated until the residual is deemed small enough.

If p_1 is in the span of the top j eigenvectors of $\mathbf{A}$, then no iteration will be necessary, and the approximation is exact. So, within our ADMM, when the span of the eigenvectors for $\mathbf{B} - \mathbf{C} + \mathbf{S}/\rho$ is similar across iterations, previous iterates can be used as initializations for the restarted Lanczos procedure. Thus, as we loop through the ADMM steps, these initializations improve the speed subsequent decompositions.

This modification significantly improves the per-iteration efficiency of the algorithm while still converging in a few dozen iterations. Note that ADMM will still converge (though in perhaps more iterations) if some or all of the steps are implemented approximately (Eckstein and Bertsekas, 1992). In particular, by approximating the projection of $\mathbf{Q}$, $\mathrm{Proj}_{\mathcal{F}^d}(\mathbf{Q})$, with $\widetilde{\mathrm{Proj}}_{\mathcal{F}^d}(\mathbf{Q})$, ADMM will converge provided

$$\sum_{k=1}^{\infty} \left\| \mathrm{Proj}_{\mathcal{F}^d}(\mathbf{Q}^{(k)}) - \widetilde{\mathrm{Proj}}_{\mathcal{F}^d}(\mathbf{Q}^{(k)}) \right\| < \infty.$$

Nishihara et al. (2015) suggests linear convergence for ADMM, and our experience is that this case remains linear despite the approximation.

6 Proofs

To prove Theorem 1, we first need the following technical lemma.

Lemma 1. *Let* $\boldsymbol{\Xi} = \mathbf{Z}\mathbf{Z}^\mathsf{T}$ *be the orthogonal projector on to the d-dimensional subspace of* $\mathbb{R}^p$ *spanned by* $\mathbf{Z}$ *with* $\mathbf{Z}^\mathsf{T}\mathbf{Z} = \mathbf{I}_d$. *Let* $b \in \mathbb{R}^n$ *and* $\mathbf{A} \in \mathbb{R}^{n\times p}$. *Then, defining* $\widehat{x} := \mathrm{argmin}_x \|\mathbf{A}\mathbf{Z}x - b\|_2^2$,

$$\begin{aligned}\mathbf{Z}\widehat{x} &= \mathbf{Z}(\mathbf{A}\mathbf{Z})^+ b = (\mathbf{A}\boldsymbol{\Xi})^+ b\\ &= \underset{y}{\mathrm{argmin}} \|\mathbf{A}\boldsymbol{\Xi}y - b\|_2^2 =: \widehat{y},\end{aligned}$$

where $\mathbf{Q}^+$ *is the Moore-Penrose generalized inverse of* $\mathbf{Q}$.

Proof of Lemma 1. We have

$$\begin{aligned}\widehat{y} &:= (\mathbf{A}\boldsymbol{\Xi})^+ b = \boldsymbol{\Xi}(\mathbf{A}\boldsymbol{\Xi})^+ b = \mathbf{Z}\mathbf{Z}^\mathsf{T}(\mathbf{A}\mathbf{Z}\mathbf{Z}^\mathsf{T})^+ b\\ &= \mathbf{Z}\mathbf{Z}^\mathsf{T}\mathbf{Z}(\mathbf{A}\mathbf{Z})^+ b = \mathbf{Z}(\mathbf{A}\mathbf{Z})^+ b =: \mathbf{Z}\widehat{x}.\end{aligned}$$

Here, the first equality is a standard property of idempotent matrices and the third follows from (Harville, 1997, Thm. 20.5.6).

Proof of Theorem 1. Write $\boldsymbol{\Pi} = \mathbf{V}_d\mathbf{V}_d^\mathsf{T}$ and $\widehat{\boldsymbol{\Pi}} = \widehat{\mathbf{V}}_d\widehat{\mathbf{V}}_d^\mathsf{T}$ for the orthogonal projectors onto the column span of the population quantity $\mathbf{V}_d$ and the estimate produced by Algorithm 2 respectively. Let

$$\tilde{\gamma} = \underset{\gamma}{\mathrm{argmin}} \|\mathbf{X}\mathbf{V}_d\gamma - Y\|_2^2,$$

and define $\tilde{\beta} = \mathbf{V}_d\tilde{\gamma}$. Note that $\beta_* \in \mathrm{col}(\mathbf{V}_d)$ and $\widehat{\beta} \in \mathrm{col}(\widehat{\mathbf{V}}_d)$ as $\beta_* = \mathbf{V}_d\mathbf{L}_d^{-1}\boldsymbol{\Lambda}_d\Theta =: \mathbf{V}_d r$ and $\widehat{\beta} = \widehat{\mathbf{V}}_d\widehat{\gamma}$. Then,

$$\begin{aligned}&\left\|\mathbf{X}(\beta_* - \widehat{\beta})\right\|_2^2\\ &= \left\|\mathbf{X}(\beta_* - \tilde{\beta})\right\|_2^2 + \left\|\mathbf{X}(\tilde{\beta} - \widehat{\beta})\right\|_2^2\\ &= \|\mathbf{X}\left(\mathbf{V}_d\gamma_* - \mathbf{V}_d\tilde{\gamma}\right)\|_2^2 + \left\|\mathbf{X}\left(\mathbf{V}_d\tilde{\gamma} - \widehat{\mathbf{V}}_d\widehat{\gamma}_d\right)\right\|_2^2\\ &= \|\mathbf{X}\mathbf{V}_d\left(\gamma_* - \tilde{\gamma}\right)\|_2^2 + \left\|\mathbf{X}\left(\boldsymbol{\Pi}\tilde{\beta} - \widehat{\boldsymbol{\Pi}}\widehat{\beta}\right)\right\|_2^2,\end{aligned}$$

where the last line follows by Lemma 1.

Now, $\mathbf{X}\widehat{\beta} = \widehat{Y} \in \mathrm{col}(X\widehat{\boldsymbol{\Pi}})$ and $\mathbf{X}\tilde{\beta} = \tilde{Y} \in \mathrm{col}(\mathbf{X}\boldsymbol{\Pi})$, and $\mathrm{col}(\mathbf{X}\boldsymbol{\Pi}) \subset \mathrm{col}(X) \cap \mathrm{row}(\boldsymbol{\Pi}) = \mathrm{col}(\mathbf{X}) \cap \mathrm{col}(\boldsymbol{\Pi})$ (because $\boldsymbol{\Pi}$ is symmetric). Thus, we have that $\widehat{Y} \in \mathrm{col}(\mathbf{X}\widehat{\boldsymbol{\Pi}}) \subset \mathrm{col}(\mathbf{X}) \cap \mathrm{col}(\widehat{\boldsymbol{\Pi}})$ and $\tilde{Y} \in \mathrm{col}(\mathbf{X}\boldsymbol{\Pi}) \subset \mathrm{col}(\mathbf{X}) \cap \mathrm{col}(\boldsymbol{\Pi})$. By linearity, there exist orthogonal

Method	Breast Cancer1		Breast Cancer2		DLBCL		AML		NSCLC	
	MSE	feature#	MSE	feature#	MSE	feature#	MSE	feature#	MSE	feature#
Ridge	0.6331	4751	0.4330	11331	0.6766	7399	2.0451	6283	0.2105	939
Random Forest	**0.5519**	4751	**0.3976**	11331	**0.6613**	7399	2.0276	6283	**0.2059**	939
SVM linear	0.6375	4751	0.4009	11331	0.7414	7399	2.5637	6283	0.2819	939
SVM RBF	0.5602	4751	0.4448	11331	0.6684	7399	**2.0106**	6283	0.2080	939

Table 6. Prediction MSE for alternative methods in real genomics data analysis.

Method	Breast cancer1		DLBCL		AML	
	Acc.%	feature #	Acc.%	feature #	Acc.%	feature #
`SuffPCR`	**60.95**	100	60.43	103	**59.39**	95
Logistic Lasso	60.00	7	56.86	5	57.57	9
Logistic Ridge	58.09	4751	**61.00**	7399	56.97	6283

Table 7. Accuracy of survival status prediction and number of selected genes for the classification tasks on real data. Results are averaged over 10 repeated iterations.

GenBank AN	description
M17886	ribosomal protein, large, P1
S79522	ribosomal protein S27a
X03342	ribosomal protein L32
D23661	ribosomal protein L37
M84711	ribosomal protein S3A
L06498	ribosomal protein S20
U14973	ribosomal protein S29
M17887	ribosomal protein, large P2
L04483	ribosomal protein S21
M64716	ribosomal protein S25
L19527	ribosomal protein L27
X66699	ribosomal protein L37a
U14970	ribosomal protein S5
X64707	ribosomal protein L13
U14971	ribosomal protein S9
M60854	ribosomal protein S16
NM_022551	ribosomal protein S18
X00452	major histocompatibility complex, class II, DQ alpha 1
X00457	major histocompatibility complex, class II, DP alpha 1
X62744	major histocompatibility complex, class II, DM alpha
U15085	major histocompatibility complex, class II, DM beta
M16276	major histocompatibility complex, class II, DQ beta 1
K01171	major histocompatibility complex, class II, DR alpha
M20430	major histocompatibility complex, class II, DR beta 5
M83664	major histocompatibility complex, class II, DP beta 1
K01144	CD74 antigen (invariant polypeptide of major histocompatibility complex, class II antigen-associated)

Table 8. A listing of the genes encoding ribosomal proteins and MHCII protein which predict DLBCL survival as selected by `SuffPCR`.

projectors $\mathbf{Q}$ and $\mathbf{R}$ such that $\widehat{Y} = \mathbf{Q}Y$ and $\tilde{Y} = \mathbf{R}Y$. Therefore,

$$\begin{aligned}\left\|\mathbf{X}\left(\mathbf{\Pi}\tilde{\beta} - \widehat{\mathbf{\Pi}}\widehat{\beta}\right)\right\|_2^2 &= \left\|\widehat{Y} - \tilde{Y}\right\|_2^2 \\ &= \|(\mathbf{Q} - \mathbf{R})Y\|_2^2 \\ &\leq \|\mathbf{Q} - \mathbf{R}\|_F^2 \|Y\|_2^2 \\ &\leq \left\|\mathbf{\Pi} - \widehat{\mathbf{\Pi}}\right\|_F^2 \|Y\|_2^2,\end{aligned}$$

where the first inequality is Hölder's inequality for the Frobenius norm. For the second, since $\mathbf{Q}$ and $\mathbf{R}$ are (orthogonal) projectors onto subspaces of col($\mathbf{\Pi}$) and col($\widehat{\mathbf{\Pi}}$), it must be that $\|\mathbf{Q} - \mathbf{R}\|_F \leq \left\|\mathbf{\Pi} - \widehat{\mathbf{\Pi}}\right\|_F$.

Now, since $\widehat{\mathbf{\Pi}}$ is a solution to the Fantope projection problem under Assumptions A1–A6, we can invoke (Vu et al., 2013, Cor. 3.3) to get that

$$\left\|\mathbf{\Pi} - \widehat{\mathbf{\Pi}}\right\|_F = \mathcal{O}_P\left(s\sqrt{\log(p)/n}\right)$$

while $\|Y\|^2/n = \mathcal{O}_P(1)$.

Turning now to $\|\mathbf{X}\mathbf{V}_d(\gamma_* - \tilde{\gamma})\|_2^2$, $\tilde{\gamma}$ is simply the ordinary least squares regression estimate under random design. Thus by, for example, (Hsu et al., 2014, Theorem 1 and subsequent remarks),

$$\|\mathbf{X}\mathbf{V}_d(\gamma_* - \tilde{\gamma})\|_2^2 = \mathcal{O}_P\left(\sigma^2 d/n\right).$$

Combining these terms gives the result.

References

J Baglama and L Reichel. Augmented implicitly restarted Lanczos bidiagonalization methods. *SIAM J. Sci. Comput.*, 27:19–42, 2005.

L Bullinger et al. Gene expression profiling identifies new subclasses and improves outcome prediction in adult myeloid leukemia. *N. Engl. J. of Med.*, 350:1605–1616, 2004.

M de Charette and R Houot. Hide or defend, the two strategies of lymphoma immune evasion: potential implications for immunotherapy. *Haematologica*, 103:1256–1268, 2018.

J Eckstein and DP Bertsekas. On the Douglas-Rachford splitting method and the proximal point algorithm for maximal monotone operators. *Math. Progam.*, 55:293–318, 1992.

SB Ednersson et al. Expression of ribosomal and actin network proteins and immunochemotherapy resistance in diffuse large B cell lymphoma patients. *Br. J. of Haematol.*, 181:770–781, 2018.

S Gnjatic et al. Seromic profiling of ovarian and pancreatic cancer. *PNAS*, 107:5088–5093, 2010.

D Harville. *Matrix Algebra From a Statistician's Perspective*. Springer, Secaucus, 1997.

D Hsu, SM Kakade, and T Zhang. Random design analysis of ridge regression. *Found. Comput. Math.*, 14:569–600, 2014.

V Lazar et al. Integrated molecular portrait of non-small cell lung cancers. *BMC Med. Genomics*, 6:53, 2013.

Y Li et al. Aberrant DNA methylation of p57KIP2 gene in the promoter region in lymphoid malignancies of B-cell phenotype. *Blood*, 100: 2572–2577, 2002.

NAP Lieberman et al. In vivo antiviral host transcriptional response to SARS-CoV-2 by viral load, sex, and age. *PLoS Biology*, 18:1–17, 09 2020.

R Liu et al. Screening of key genes associated with R-CHOP immunochemotherapy and construction of a prognostic risk model in diffuse large B-cell lymphoma. *Mol. Med. Rep.*, 20:3679–3690, 2019.

NI Lorè et al. CXCL10 levels at hospital admission predict COVID-19 outcome: Hierarchical assessment of 53 putative inflammatory biomarkers in an observational study. *Mol. Med.*, 27:1–10, 2021.

Y Miao et al. Genetic alterations and their clinical implications in DLBCL. *Nat. Rev. Clin. Oncol.*, 16:634–652, 2019.

LD Miller et al. An expression signature for p53 status in human breast cancer predicts mutation status, transcriptional effects, and patient survival. *PNAS*, 102:13550–13555, 2005.

R Nishihara et al. A general analysis of the convergence of ADMM. In *ICML*, volume 37, pages 343–352. PMLR, 2015.

RL Novak et al. Gene expression profiling and candidate gene resequencing identifies pathways and mutations important for malignant transformation caused by leukemogenic fusion genes. *Exp. Hematol.*, 40:1016–1027, 2012.

A Rosenwald et al. The use of molecular profiling to predict survival after chemotherapy for diffuse large-B-cell lymphoma. *N. Engl. J. Med.*, 346: 1937–1947, 2002.

LJ Van't Veer et al. Gene expression profiling predicts clinical outcome of breast cancer. *Nature*, 415:530, 2002.

VQ Vu, J Cho, J Lei, and K Rohe. Fantope projection and selection: A near-optimal convex relaxation of sparse PCA. In *NeurIPS*, pages 2670–2678, 2013.

MG Waugh. Amplification of chromosome 1q genes encoding the phosphoinositide signalling enzymes PI4KB, AKT3, PIP5K1A and PI3KC2B in breast cancer. *J. Cancer*, 5:790—796, 2014.

W Zhang et al. COVID19db: A comprehensive database platform to discover potential drugs and targets of COVID-19 at whole transcriptomic scale. *Nucleic Acids Res.*, 50:D747–D757, 2021.

Y Zhou et al. Functional dependency analysis identifies potential druggable targets in acute myeloid leukemia. *Cancers*, 12:3710, 2020.
